# The treatment action campaign and the three dimensions of lawyering: Reflections from the rainbow nation

**DOI:** 10.1080/17290376.2013.807067

**Published:** 2013-07-02

**Authors:** Hassan Ahmad

**Keywords:** HIV/AIDS, Treatment Action Campaign, South Africa, PAHRO, activism, VIH/SIDA, Treatment Action Campaign, Afrique du Sudentravait, PAHRO, activisme

## Abstract

The spread and perpetuation of the HIV/AIDS epidemic in South Africa has hindered the country's social and economic growth after apartheid. This paper documents my experiences while working with the Projects Abroad Human Rights Office and specifically my interactions with the Treatment Action Campaign (TAC), an organization which has taken a multi-dimensional approach in order to educate people about HIV/AIDS and attempt to provide access to medicines for millions of South Africans afflicted with the disease. I discuss how TAC has used both traditional and non-traditional methods of advocacy to combat the epidemic and equate access to health care to a social justice issue by empowering marginalized communities. The paper's dual purpose is to applaud TAC's continuous success in combating HIV/AIDS with such a multi-dimensional approach and illustrate how other organizations can utilize such an approach in order to affect social change. To illustrate TAC's approach, I utilize Lucie White's three dimensions of lawyering and equate TAC to a single cause lawyer, signifying that White's characterization of multi-dimensional activism is not limited to individuals, but can rather be applied at the firm level. White's three dimensions include: (a) advocacy through litigation, (b) advocacy in stimulating progressive change, and (c) advocacy as a pedagogic process. From this analysis, I conclude that TAC's multi-dimensional approach and specifically its inherent practice of White's three dimensions has been the root of its success in educating millions about the virus and advocating for access to medicines for those who have contracted HIV. TAC's innovative advocacy has also mobilized a new generation of South African activists who have helped TAC grow into a vibrant and integral organization within the country's post-apartheid culture. Such an example can serve as a framework for future organizations who wish to tackle other challenges that face the country.

## Abstract (FR)

L'expansionet la perpetuation de l'épidémie du VIH/SIDA en Afrique du Sudentravait la croissancesociale et économique du pays après l'apartheid. Cet article documentemesexpériences en travaillant avec le bureau des droits de l'homme et des projets à l'étranger (Projects Abroad Human Rights Office, PAHRO) et particulièrementmes interactions avec la Treatment Action Campaign (TAC), un organisme qui a adoptéuneapprochemultidimensionnelleafind'éduquer les gens sur le VIH/SIDA et tenter de fournirl'accès aux médicaments pour des millions du Sud-Africains qui souffrent de la maladie. Je discute comment la TAC a utilisé des méthodestraditionnelleset non traditionnelles de plaidoyer pour s'opposercontrel'épidémie et j'assimilel'accès aux soins de santé à une question de justice sociale en responsabilisant les communautésmarginalisées. Le double objectif de cetteétudeestd'applaudir le succèscontinu de la TAC dans la luttecontre le VIH/SIDA avec uneapprochemultidimensionnelle et d'illustrer la façondontd'autresorganismespeuventutiliserunetelleapprochedans le but d'influersur le changement social. Pour illustrerl'approche de la TAC, j'utilise les trois dimensions de la pratique du droit de Lucie White et assimile TAC à uneseuleavocatece qui signifieque la caractérisation de l'activismemultidimensionnellene se limite pas aux individus, maispeutêtreappliquée au niveau des entreprises. Les trois dimensions de White sont: (a) le plaidoyerpar la voie du litige (b) le plaidoyer pour stimuler le changementprogressifet (c) le plaidoyercomme un processuspédagogique. De cette analyse, je conclusquel'approchemultidimensionnelle de la TAC etparticulièrementsapratiqueinhérente des trois dimensions de White ontété la racine de son succèsdansl'éducation de millions sur le virus et le plaidoyer pour l'accès aux médicaments pour les personnes qui ontcontracté le VIH. Le plaidoyerinnovant de la TAC a égalementmobiliséune nouvelle générationd'activistessud-africains qui ontaidé le TAC à se transformer en unorganismedynamique et essentiel au sein de la culture après l'apartheid du pays. Untelexemplepeutservir de cadre pour les futures organismessouhaitant à releverd'autresdéfis qui se posent au pays.

## Introduction

Sitting in the back of the car looking out at Cape Town's picturesque landscape amidst ancient mountains, which undoubtedly served as witnesses to countless European incursions culminating in subsequent Dutch and British colonialism, I could not help but feel the curiosity of a young child, eager to learn of its surroundings in an unfamiliar setting. My driver, the social outreach coordinator at the Projects Abroad Human Rights Office (PAHRO), served as a liaison connecting me with various HIV/AIDS organizations actively working to eradicate the almost 20-year long epidemic plaguing South Africa and killing approximately 300,000 people per year in the country, a rate of approximately 900 deaths per day (‘Mid-Year Population Estimates’ 2009, http://www.statssa.gov.za/publications). As we passed the Groote Schuur hospital, the teaching hospital for the University of Cape Town's faculty of medicine (Harrison 2004), the country's advancement seemed apparent and even reminiscent of western countries. However, my notion to compare South Africa with the likes of Europe or North America quickly faded as we proceeded onto the highway distancing ourselves from the city centre. I witnessed, in astonishment, endless waves of makeshift shacks, pieced together from spare metal and wood, standing awkwardly, inherently symbolizing their fragility. It was the very phenomenon about which I had heard before landing in Cape Town, namely of the innumerable townships,^1^ home to South Africa's historically marginalized black – and even at times white – communities, still recovering from the apartheid era.

We were heading to Khayelitsha, Cape Town's largest township and home to over 1.25 million residents, composed of both native South Africans and refugees from neighbouring African countries such as Botswana, Namibia and Zimbabwe (‘The Population Register Update: Khayelitsha’ 2006, http://www.capegateway.gov.za). We had scheduled a meeting at the Treatment Action Campaign (TAC) (‘Resource for Health Campaign’, http://www.tac.org.za/community) field office to discuss the organization's work and possible assistance with surveys assessing Khayelitsha residents’ current access to anti-retroviral (ARV) treatment (‘Guidelines for Anti-retroviral therapy in Adults’ 2006, http://localhost:81/sahivsoc) and general viewpoints regarding the government's role in combating HIV/AIDS. We were scheduled to meet with a TAC employee and activist who worked to co-ordinate initiatives between health clinics in Khayelitsha and TAC's regional office. This particular TAC employee had valuable contacts within Khayelitsha's healthcare community, an essential requirement if we were going to be granted allowance to conduct surveys in clinics, a privilege that would otherwise require a lengthy authorization process taking several weeks or even months.

As we continued along the highway gazing through the welcomed protection of our automobile windows, we were separated not only by physical distance from the perpetual suffering of township dwellers, but also by the metaphysical barriers of class, privilege and opportunity. We noticed only minute distinctions between adjacent townships, including occasional advertisements or peculiar informal antennas, serving as technological anachronisms attempting to bridge the socio-economic gap ever apparent in South African society. Our PAHRO guide told us the history behind the townships and how they were deemed ‘black-only’ land designations during apartheid, separated from metropolitan city centres inhabited by the minority white and coloured communities. I was informed concerning the swelling of township populations after mass immigration from the Eastern Cape due to superior public health and education services in the Western Cape. Despite witnessing horrid living conditions throughout the car ride, I was convinced that the TAC office would be more reminiscent of a western-styled NGO, teeming with structure and organization, business-like and professional. I maintained this notion in light of TAC's international acclaim following its decade long record of activism that had brought the South African government and powerful international pharmaceutical companies, such as Pfizer, to their knees.

Upon arriving in Khayelitsha, in a crowded marketplace filled with minibus taxis and blaring reggae and rap music, overshadowing sporadic cries in Xhosa and dialectical English, I spotted the TAC emblem signifying the office's entrance. As we climbed the stairs, passing the *Medicines Sans Frontieres* (*MSF*) office housed in the same building, my pre-conceived notions of a setting with all the luxuries befitting the bureaucracy and privilege to which I was accustomed quickly faded. I soon realized that I was about to be introduced to the organization that had mobilized a new generation of South African AIDS activists to attempt ending the epidemic that had claimed so many of their generation.

As our contact at TAC was running behind schedule, our PAHRO guide, another PAHRO volunteer and I waited patiently in the crammed common area while gazing at volunteers hurrying throughout the office and simultaneously speaking about current initiatives and the day's activities in a non-chalante, jovial demeanour not unknown to indigenous South Africans. In those fifteen minutes while waiting for the TAC employee, I learned what so many visitors from around the world had recognized about TAC. The organization's inimitability stemmed from its grassroots mobilization efforts and ability to utilize local talent to educate the traditionally marginalized black South African community about HIV/AIDS. TAC has sustained this endeavour by going beyond the traditional spheres of advocacy and using innovative approaches to enable social change. It has employed a nurturing approach centred on sympathy and compassion rather than judgment and antagonism.

This paper will discuss how TAC has used both traditional and non-traditional methods of advocacy in South Africa to combat the HIV epidemic and equate access to health care to a social justice issue by empowering marginalized communities. TAC, in endeavouring to raise the social conscience of South Africans in understanding the destructive nature of the HIV/AIDS epidemic, has used but has not limited itself to the county's legal and political institutions. One purpose of this paper is simply to describe and applaud TAC's continuous success in combating the spread of HIV/AIDS and the organization's growing influence in post-apartheid South Africa. Another purpose though is to delineate TAC's multi-dimensional approach and how other similar organizations within South Africa and abroad can utilize such a model in order to affect social change. To illustrate TAC's successful strategy of promoting social change, I will employ Lucie White's three dimensions of lawyering (White 1988) and discuss how TAC has unequivocally succeeded at each individual dimension. In this discussion, I equate TAC, an advocacy organization, to a single cause lawyer signifying that White's three-dimensional analysis can be applied at both the individual and firm level in order to analyse multi-faceted approaches to promoting social change. As I proceed through the discussion, I will inject my own experiences from my summer 2010 fellowship in Cape Town with PAHRO in which I frequented TAC's Khayelitsha office, worked with TAC representatives to conduct surveys in township clinics and researched TAC's legal claims for a study elucidating inequalities in South Africa's two-tiered healthcare system.

## TAC and the three dimensions of lawyering

TAC was established on International Human Rights Day on 10 December 1998. On that day, a group of approximately fifteen people protested outside the St. Georges Cathedral in Cape Town demanding treatment for people living with HIV (Robins & von Lieres 2002). At the time, almost three million people in South Africa lived with the virus (‘AIDS in South Africa’ 1998, http://www.pbs.org/newshour), a figure that had reached 5.2 million by mid-year 2009. Just as the number of South Africans living with HIV swelled, so too did TAC's numbers, which as of March 2007 had almost reached 20,000 volunteers and workers throughout the country with six provincial, nine district and 200 branch offices (Peacock, Budaza, & Greig 2008). TAC's uniqueness and simultaneously its foundation for success, however, stems from its bottom-up approach which empowers traditionally marginalized segments of South African society by mobilizing them to lead, educate and organize their own people to make a positive change and stand up for their legal rights. TAC's concurrent operations at the local, national and global levels have granted it legitimacy in rural township homes, board rooms and court houses across South Africa and abroad.

As the three of us continued to wait for our contact at TAC, I increasingly realized the importance of cultural legitimacy when attempting to empower people concerning their legal rights. The melodic clicking and humming of Xhosa filled the common area interrupted by sprinkles of English, re-iterating the pride Khayelitshans have in their ancestral roots. As we were about to depart, convinced that our TAC contact would not be able to meet with us, a young woman walked in, unlikely out of her 20s, with the attire and accessories of a modern college student while also exuding an intangible sense of authentic African culture. Our contact, although differing vastly from my preconception of how a professional in her position would appear, fitted TAC's approach to mobilizing disempowered people and promoting social change in South Africa. Fluently discussing political matters and possible obstacles that may arise in conducting surveys, she represented a new generation of South African youth, who were sufficiently ingrained in their cultural upbringing while concurrently maintaining a firm grasp of modern technology and society to efficiently work within the new global ethos. After our brief discussion, I could not help but think what a marginal role such a person in her position would have played had apartheid still existed or, moreover, had TAC not existed.

Lucie White, in her article entitled *To Teach and Learn: Lessons from Driefontein on Lawyering and Power*, related a 1985 story about Driefontein, a predominantly black village in the northern part of South Africa, which was located in a white designated area. White elucidated how a lawyer and an organizer, with varying skills and methods, empowered the villagers to mobilize and dissent against the forced removal, a strategy proven successful upon the villagers' allowance to remain in their previous dwellings. White argued that an advocacy method focused on social change, which comprehends that subordination is imprinted onto the psyches of disenfranchised clients should focus on empowering those clients rather than imposing socio-legal solutions. White posited that the former is a more effective method of achieving sustainable emancipation.

White pairs her three dimensions of lawyering to Steven Lukes' three dimensions of subordination (Lukes 2005) and conceives that each subsequent level of subordination elicits a dimension of lawyering. In the following sections, I will discuss how TAC has systematically understood the various levels of subordination and employed all of White's three dimensions of lawyering to mobilize traditionally marginalized societal segments in the process of self-empowerment and education to combat the HIV/AIDS epidemic. Although both the dimensions of lawyering and levels of subordination are progressive such that each subsequent dimension or level is more intense than previous dimensions or levels, I will elucidate how TAC is employing a multi-dimensional approach that takes into account all three dimensions of lawyering and levels or subordination, thus fulfilling White's optimal method of advocacy that promotes efficient and sustainable internal empowerment leading to social change.

## First dimension: advocacy through litigation

White entitles the first dimension of lawyering as ‘The Contest of Litigation’ and says that in this dimension the lawyer ‘is charged with designing and winning lawsuits that will further the substantive interests of client groups. The lawyer “translates” client grievances into legal claims’. White exclaims that it is not the lawyer's role in this dimension to question the structure of the law or the judicial system, but rather to effectively litigate in a previously established power structure that has inherently created class, race and gender imbalances. This first dimension of lawyering correlates to Lukes’ first level of subordination, which he classifies as ‘Interest Group Contestation’. At this level, power is exercised by contesting interests through established political and legal channels. In Lukes' framework, a group gains power at this first level when it successfully prevails at a particular contest.

TAC has utilized the established legal and political avenues in South Africa to disseminate its message about the HIV epidemic and challenge injustices that limit access to treatment, particularly for the marginalized poor. TAC's 2002 claim against South Africa's Minister of Health was an example of White's first dimension of lawyering (*Treatment Action Campaign v. Minister of Health*, 2002). In that case, TAC successfully sued the government for not guaranteeing that prevention of mother-to-child transmission through the drug nevirapine was available in public hospitals, thus contravening Section 27(1)(a) of the South African Constitution guaranteeing every citizen a right to have access to healthcare services (Constitution of the Republic of South Africa 1996). Chaskalson CJ, in his decision, stated, ‘We do not underestimate the nature and extent of the problem facing government in its fight to combat HIV/AIDS and, in particular, to reduce the transmission of HIV from mother to child … But the nature of the problem is such that it demands urgent attention. Nevirapine is a potentially lifesaving drug’ (*TAC v. Minister of Health*). Due to TAC's activism in challenging what was then the government's policy and the court's subsequent decision to order South African hospitals to provide nevirapine to expecting and new mothers, innumerable children who would otherwise have been afflicted with paediatric HIV have not inherited the virus from their mothers.

Another example of TAC's utilization of White's first dimension of lawyering is its 2009 lawsuit against the Department of Correctional Services (DCS) demanding access to treatment for HIV infected prisoners (*Treatment Action Campaign v. Minister of Correctional Services*, 2009). In that case, TAC pressured the Minister of Correctional Services to hand over a report outlining the cause of death of an inmate at the Westville Correctional Services (WCC). Following a production order from the court, the WCC submitted the inmate's full report upon which time it was discovered that lack of access to ARVs contributed to the inmate's death. The DCS was ordered to comply with Section 27 of the South African Constitution by providing ARVs to its HIV inmates. Furthermore, both TAC and the AIDS Law Project remained engaged in the matter by staging acts of civil disobedience by occupying Cape Town's Human Rights Commission offices to force the DCS to comply with the court ruling.

As White notes, the first dimension of lawyering seeks to ‘redistribute power to subordinated groups’. In each of the above examples, TAC's successful legal claims benefited disempowered groups, whether it was mothers and their newborn children or prisoners in a correctional facility. Rather than question possible biases or injustices inherent in the legal system, TAC utilized the established avenues in attempting to rectify an injustice enacted by the dominant paradigm against a marginalized subsection of society. Moreover, in accordance with Lukes' first level of subordination, TAC gained power and influence with each subsequent legal victory. In successfully challenging government structures, TAC put the rule of law – through which imbalances of power could be remedied if the government was not adequately fulfilling its citizens’ rights to provide access to health care – into practice.

Despite its legal successes, TAC did not limit its advocacy to previously established avenues of justice but rather employed a multi-dimensional approach that engaged prior and new supporters outside of the courtroom, whether in seminar rooms, corporate offices or street corners. In the following sections, I discuss how TAC's approach has been an ideal illustration of White's second and third dimensions of lawyering.

## Second dimension: advocacy in stimulating progressive change

White entitles her second dimension of lawyering ‘Law as a Public Conversation’. She exclaims that in this dimension ‘the lawyer acknowledges that litigation can sometimes work directly to change the allocation of social power. However, she sees these effects as secondary to law's deeper function in stimulating *progressive change*‘. In this dimension, White acknowledges that the legal process has a role in shaping society's fabric. The law not only comprises punitive measures but also has political implications that contribute to power dynamics between the various social, political and economic groups within a developing society. This latter function is amplified in South Africa, especially given the country's history of apartheid and systemic marginalization. White continues by noting that, in this dimension, law is regarded as more than a contestation in which success is denoted by courtroom victory. Congruous to White's second dimension of lawyering is Lukes' second level of subordination, which enhances the first level that looks specifically at contests that *are* fought. This level delves into the causes for suppression of a particular conflict. In other words, the second level scrutinizes the barriers that inhibit certain issues from being challenged. These barriers can be explicit legal exclusions, a threat of retaliation, institutional restrictions, cultural idiosyncrasies or lack of legitimacy on the part of a subordinate group.

In its short history, TAC has utilized the adversarial legal system to promote social progression by pursuing already enshrined rights while also challenging unjust norms to achieve further rights for HIV patients. Exemplary of TAC's unwavering adherence to this dimension of lawyering was the Christopher Moraka Defiance Campaign. In that instance, the Pharmaceutical Manufacturers Association (PMA) challenged the government's right pursuant to the *Controlled Medicines and Related Substances Act* to lower the price of fluconazole, a brand name drug manufactured by Pfizer. Fluconazole, an ARV treatment scientifically proven to combat the opportunistic infections caused by the HIV virus was, previous to the act, unavailable through the public healthcare system (*Pharmaceutical Manufacturers Association of South Africa and Another: In re Ex Parte President of the Republic of South Africa and Others*, 2000). TAC acted as amicus curiae in the case by submitting a brief in favour of the government's position to lower the price of fluconazole making it affordable to more HIV patients. In its submission, TAC raised three issues: access to essential medicines, patent rights and health service transformation (Jara 1999).

In this instance, TAC utilized established institutions for more than simply a legal victory. TAC's role as amicus curiae and advocate for affordable medicines elaborated upon social issues throughout the campaign that were brought to light by Christopher Moraka's death. One salient issue was the inequality in the two-tiered South African healthcare system in which the private sector accounted for 75% of public expenditures on health care while serving only 20% of the population. Visiting numerous public and private clinics in addition to state run hospitals, I became accustomed to this ubiquitous reality. Hundreds of patients waiting to see one rotating physician at eight o'clock in the morning in an old, cramped public clinic far overdue for repairs became a normal sight. Conversely, I witnessed nearly empty waiting rooms serviced by sometimes five to six physicians working in a private clinic. Upon asking one private practitioner about the apparent disparity between sectors in his profession, he responded by acknowledging grave deficiencies, but then immediately remarked that if he were to transfer his practice to the public sector he would have to work longer hours, arrive at his luxury home in Green Point (along the ocean in Cape Town) after dinnertime and not be able to send his ‘princesses’ to a superior private school. Judged through the lens of South African law, the private practitioner cannot be faulted. His aloofness to the crisis signifies a greater social crisis that TAC was attempting to illuminate through the Defiance Campaign, namely the superiority of private health care in South Africa, which attracts more doctors and public funding to sustain advanced facilities that cater to the wealthy and, most often, minority white and coloured populations (Coovadia, Jewkes, Barron, Sanders & McIntyre 2009; Department of Health 1997; Ataguba 2010; Harrison 2010; McIntyre & Thiede 2007; Van der Berg, Burger, Theron, Venter, Erasmus & van Eeden 2010). As some academic observers of the Rainbow Nation have noted, health care has secondary manifestations that extend beyond scientific endeavour or disease management to the promotion and perpetuation of racial and ethnic inequalities remaining from the apartheid era.

TAC's submission also pointed to the archaic patent law framework within South Africa that was preserving unnecessary monopolies thus maintaining high drug costs, which prevented essential medicines from reaching public sector patients. The patent regime, similar to the resource and funding allocation disparities between the public and private healthcare systems, was creating an inequality that kept previously marginalized groups disempowered by inhibiting their improvement in health care. Additionally, as Lukes' second level of subordination posits, legal exclusions – patents in this instance – were acting as barriers to equal access to a public good. Furthermore, such barriers were given legitimacy since they were codified in South African patent law. Another possible barrier in Lukes' framework preventing social inequities from being challenged was the threat of retaliation. In this example, if the pharmaceutical companies’ patent rights were challenged, the fear existed that they would move their investment elsewhere dissatisfied by not being able to sell their drugs at desired premiums. TAC's Defiance Campaign and participation in the law suit challenged the established power structure in which the pharmaceutical companies were previously immune to their business decisions being questioned. Throughout the Defiance Campaign, TAC, utilizing print and digital media, was able to illuminate unjust patent right provisions being invoked by the PMA in challenging the *Controlled Medicines and Related Substances Act.* Thus, TAC was able to penetrate previously existing barriers resulting from power imbalances to promote social progress.

In the submission, the then TAC national chairperson Mazibuko Jara wrote:

The diversion of scarce resources to care for people with HIV, will very rapidly impact on other areas of public health and on the overall quality of medicine and care in South Africa. This will divert money away from key aspects of health service transformation such as equipping primary health care clinics, training nurses and doctors and improving access to HIV testing and counseling. You [PMA] have the means to contribute to preventing this.

TAC recognized that the issue of high mark-up prices on flucanazole was not an isolated incident but rather a significant injustice affecting the entire healthcare system. If the government were compelled to purchase flucanazole at exorbitant rates from international pharmaceutical companies, it would be further stretching an already depleted public healthcare system thus being unable to sufficiently prevent other opportunistic diseases caused by HIV, such as Meningitis or Tuberculosis. This would, in turn, contribute to the already apparent disparity in health care propagating the subordination and marginalization of public healthcare consumers. In fulfilling White's second dimension of lawyering, TAC comprehended the adverse social implications to the public healthcare system in the event of a successful PMA claim. It thus utilized the law as a shield against continued corporate hegemony and a vehicle for social progress.

Tilly remarks that for a cause to become a movement it requires ‘a sustained series of interactions between power holders and persons successfully claiming to speak on behalf of a constituency lacking formal representation’ (Sarat & Scheingold 2006). In this second dimension, TAC used a legal claim to stimulate a progressive movement or *change.* It fully engaged powerful pharmaceutical companies in speaking on behalf of HIV patients who previously did not have access to essential medicines. TAC sought legal avenues to change the dynamic between various socio-economic groups in South African society and bridge agelong inequalities between the formal employment sector that was privileged to have access to medical schemes (*Medical Schemes Act*, 1998) subsidizing health care and the informal sector, which relied on the grossly inadequate and underfunded public system. As a result of TAC's pressure, Pfizer decided to supply fluconazole free to public healthcare clinics in South Africa in March 2001. The Christopher Moraka Defiance Campaign proved successful as the PMA decided to withdraw its legal challenge against the government, thus allowing the latter to reduce the price of medicines imperative to the well-being of HIV patients.

## Third dimension: advocacy as a pedagogic process

White entitles her third dimension of lawyering ‘Lawyering Together Towards Change’. Using Paulo Friere's works on critical consciousness, White categorizes this dimension as a pedagogic process which is an ‘unconventional, non-hierarchical learning practice in which small groups reflect together upon the immediate conditions of their lives’. White emphasizes that this dimension is a continual process of reflection, action and improvement in which a disempowered group strives to recognize and collectively change its present reality. This continuous process fits together with Lukes' third level of subordination in which the analysis focuses on the social processes which reinforce a subordinate group's position. According to Lukes, the subordinate group is socialized into the norms and practises of the dominant group, numbed to any notion that change is, or should be, possible. Additionally, Lukes remarks that a repeated dichotomy of domination and defeat engenders a psychological withdrawal of the subordinate group from the public arena, leading to a sense of fatalism, self-deprecation, lethargy and disunity.

The already compromised state of the public healthcare system in South Africa was further encumbered by the HIV epidemic, leaving the poor majority who rely on the public system psychologically defeated and apathetic to its position vis-à-vis the private system, which was mainly utilized by the minority white population during and after apartheid. In this light, TAC's approach to HIV awareness and education at a grassroots level about citizens' legal rights and methods in which to mobilize to cause social change has sparked a reflective process, especially within rural townships (Friedman & Mottiar 2005). TAC has established AIDS literacy campaigns in townships across South Africa challenging previously accepted AIDS myths, rejection and misinformation. As Robins and von Lieres note, the struggle for access to treatment for people living with HIV/AIDS is a story about new forms of citizenship. Thus, TAC is facilitating these new forms of citizenship by emphasizing active participation within society centred upon objective reflection and methodological yet passionate action. Specifically, according to Robins and von Lieres, TAC has utilized two methods to prompt these new forms of citizenship: (i) acting as an intermediary institution serving to interface between the state and the poor; and (ii) promoting non-institutional forms of citizenship created by marginalized people themselves (Robins & von Lieres 2004).

An example of TAC's promotion of the first form of citizenship is its Resource for Health Campaign (RHC), which is interfacing the government with HIV patients lacking access to treatment. The RHC's focus is on meeting the National Strategic Plan (NSP) goals set by the government of South Africa in 2007, namely to reduce the rate of new HIV infections annually by 50% and provide ARVs to 80% of those requiring them by 2011. As Tempelman and Vermeer remark, the NSP, despite being ambitious, is a clear strategy attempting to address salient issues posed by HIV and AIDS to the social, cultural and economic development, progress and stability of all sectors within South Africa (Tempelman & Vermeer 2006). Despite the government being far from reaching its desired NSP goals, TAC's national and international recognition and legitimacy within townships and marginalized communities are more than adequate support. TAC states that the goals of the RHC are (i) to demand early treatment of infants and essential medical supplies for PMCTC; (ii) to treat patients at a 350 CD4 count, rather than 200 and eradicate ARV waiting lists; and (iii) to fight for integration of TB/HIV treatments (http://www.tac.org.za/community/node/2742). Both the NSP and RHC's goals exemplify TAC's role as an intermediary institution as they simultaneously attempt to improve access to treatment by lobbying the government to change its policies regarding access to ARVs and educate disenfranchised groups such as black youth, women and township dwellers about the importance of safe sexual practices that will inevitably decrease the annual HIV prevalence rate by 50%. Thus, TAC's multi-dimensional and methodical grassroots approach that appreciates the ethnic sensitivities of the past is crossing conventional political boundaries by waging battles both at the governmental level and in the streets.

The Christopher Moraka Defiance Campaign, introduced above, is another example of the TAC's promotion of participatory citizenship by acting as an intermediary between the State and the poor. TAC instituted a stellar media campaign to educate poor South Africans about the injustices of generic drug price increases. Additionally, TAC held multi-racial, multi-class demonstrations and workshops in collaboration with international NGOs such as *MSF* to mobilize the poor and assist the government in its claim against the PMA. Upon discontinuing its case, the PMA remarked on the impetus for the withdrawal by pointing to the adverse publicity received against it and the multinational pharmaceutical companies which it represents as a result of TAC's accusations that corporate greed was triumphing over public health. Never before in recent history had a South African grassroots organization proven so successful in its lobbying strategy against a financially superior entity. TAC had mobilized those directly affected by the HIV virus to protest in large numbers against an injustice denying the poor access to essential medicines that would prolong their lives with HIV. TAC was able to mobilize various groups spanning the socio-economic spectrum to support the government's right to lower the price of essential medicines. In pursuing such an endeavour, TAC exemplified its role in promoting a novel form of South African citizenship centred upon activism and responsible government. Moreover, TAC played the role of a catalyst in intervening between the government and marginalized communities who were seeking increased access to treatment.

Subsequent to the Defiance Campaign, but also keeping in line with TAC's role as an intermediary institution, its December 2001 appearance before the High Court of South Africa claimed that the government had a positive obligation, subsequent to Section 27(2) of the South African Constitution, to promote access to health care and thus access to HIV/AIDS drug treatment. TAC lawyers emphasized citizen rights to health care and the socio-economic consequences of not recognizing the government's positive obligation in Section 27(2). In addition to agreeing that Section 27(2) elicits such an obligation, the court addressed the continuing scientific debate looming in South Africa at the time about the legitimacy of the causation between HIV and AIDS, concluding that an interrelationship does exist. TAC's activism eventually led to the 2003 National ARV Treatment Programme, at which time ARV treatment became fully subsidized by the government in the public healthcare system.

From the above examples, TAC bridged the gap between established state apparatuses and traditionally disenfranchised South Africans empowering the latter to fully realize their rights in the post-apartheid system.

The second form of citizenship TAC has promoted is non-institutional and created by marginalized people themselves. In this light, TAC has successfully mirrored previous social movements centred on health issues; a concept now termed ‘biological citizenship’ (Petryna 2002). Michel Foucault referred to this phenomenon as the ‘microphysics of power’, which yields the dispersal of fluxes of power through all the crevices of the social system and the propagation of knowledge, practices, meaning and identities from the deployment of power (Epstein 1996). Various examples illustrate TAC's promotion of self-empowerment leading to marginalized groups mobilizing themselves. In 2004, after the rape and murder of TAC activist Lorna Mlofana at a Khayelitsha shebeen, residents in the rural township came together to form two organizations for men and women encouraging open dialogue about gender-based discrimination and abuse and what action would be required to help mitigate the phenomenon. From this activism stemmed Positive Women United (POWU) and Positive Men United (POMU), both of whom were supported and promoted by TAC. Mandla Majola, a POMU member, described the development of such structures by saying, ‘Men struggle to find their dignity in Khayelitsha. We have high unemployment. So many men feel useless. They use alcohol and drugs to try to cure their frustrations. Then they vent their anger on women because they think women won't fight back. We need to give men their dignity back’ (Kamkam & Geffen 2006).

Another example of TAC's promotion of non-institutional mobilization of marginalized groups is its support of the Thatnusizo Support Group in Inanda, Durban. The group originally began with only three people, all of whom had lost children to AIDS. Empowered by TAC workshops and educational material combating the stigma behind HIV/AIDS, the group grew by knocking on doors and spreading information to neighbours. As one TAC volunteer noted about TAC's role in transforming people from passive subjects into active participants, ‘We were a top-down organization but now our members are showing a potential to lead. Branch members used to just keep quiet in meetings, but are beginning to participate actively – it started small but then it grew’ (Friedman & Mottiar 2004). In this manner, TAC has revived a long dormant sense of activism and citizenship by supporting groups of people looking to start their own initiatives in attempting to eradicate HIV/AIDS.

During my time in Cape Town, I read about TAC's intermediary role in bridging the gap between the impoverished majority and State institutions. However, it was the second form of participatory citizenship, namely the promotion of non-institutionalized forms of public engagement that I witnessed for myself. TAC representatives from its Literacy and Treatment Program (LTP) visited Khayelitsha clinics, which were no more than modest building structures with usually one or two healthcare workers and rarely a physician, three times weekly to give presentations to the long line of patients waiting for their clinical appointments about the importance of safe sexual practices and regular adherence to treatment schedules. The LTP volunteers, themselves residents of Khayelitsha, achieved an immediate legitimacy when speaking with patients and residents after their presentations about personal, social and health-related matters. This legitimacy, rooted in a cultural and historical congruency, would be difficult to attain for an outsider such as myself seeking to further understand the myriad of socio-economic issues pertaining to the HIV/AIDS epidemic. TAC's grassroots strategy has empowered their volunteers to consider their work with a sense of responsibility and ownership that inevitably will lead to greater prosperity for the volunteers' own communities. Whereas the government and external international organizations have failed to gain this respect and legitimacy due to a history of mistrust and adversarial approaches, TAC has sought to personalize the HIV/AIDS virus to the marginalized communities which it afflicts. Due to this approach, TAC is invigorating the next generation of activists to seek a better future for South Africa while breaking the shackles of the past.

By simultaneously promoting both forms of the citizenship discussed above, TAC has engaged White's pedagogical process by encouraging public health care consumers originating from marginalized communities to reflect upon their circumstances and seek to improve their lives through organized and dedicated action. Likewise, TAC has revived such marginalized groups from Lukes' third level of subordination which manifests in the form of fatalism and psychological withdrawal by educating the masses about their newfound position in the post-apartheid framework in which they can compete on an equal basis despite ethnic and racial differences. Although much work is required in the future for full emancipation and equality, TAC's approach, which allows communities to forge solutions for themselves rather than relying on external and, often times, dominating forces, is promoting sustainable social progress already manifesting in greater mobilization, confidence, entrepreneurship and higher standards of living.

## Conclusion

The foregoing discussion illustrated how TAC's grassroots approach has exemplified Lucie White's three dimensions of lawyering. Throughout its history TAC has approached advocacy through litigation, by attempting to stimulate social change and by engaging in a pedagogical process. In this process, TAC has acknowledged the deeply ingrained sense of subordination among South Africa's majority black population, which remained suppressed through government policies and legislation during apartheid. Although the formal era of apartheid has ended, a symbolic apartheid continues to exist in which there is unequal access to essential resources, especially health care. HIV continues to ravage the South African population with approximately 17% of 15–49 year olds being HIV positive. I witnessed the effects of such a statistic firsthand upon visiting an HIV orphanage in Masiphumelele, a rural township approximately 45 minutes outside of Cape Town. As I conversed and played with the children, full of life, laughter and hope far beyond the expectations of an external observer viewing their circumstances, the orphanage's director informed me that many of the children had contracted HIV through MTCT. However, she followed her statement by remarking that all of the children were completely healthy due to sufficient access to ARV treatments. As we sat in the director's office afterwards, energized by our foregoing activities with the children, I felt invigorated by the possibilities available to all South African HIV patients upon the realization of the 2011 NSP goals. This realization of goals would raise hope for South Africans to realize a better future for itself based on the opening provision of its constitution which states that, ‘The Republic of South Africa is one, sovereign, democratic state founded on the … values [of] human dignity, the achievement of equality and the advancement of human rights and freedoms.’ Although the normative provisions of the South African Constitution stand in contrast to omnipresent social and economic realities, TAC's innovative advocacy throughout the past decade has mobilized a new generation of South Africans to participate fully in all aspects of society to combat injustices perpetuating the HIV epidemic. TAC's effect on society spans further than health care and challenges the legitimacy upon which the apartheid regime was constructed. Its multi-dimensional approach, illustrated in this paper through White's dimensions of lawyering, has demonstrated the law's ability to transform society when viewed through the goal of social progress. Invigorated by the possibilities open to the Rainbow Nation, I walked out of the director's office with a greater sense of purpose in my own practice of law to facilitate social change. From this feeling, as I left the orphanage, I could not help but recite the lyrics from the theme song of the 2010 Soccer World Cup, ‘When I get older, I will be stronger, they'll call me freedom, just like a waving flag …’ (K'naan 2009).

## Note

1. Township is a synonym for slum. However, the former is used more often in South Africa.
